# Determinants of Postpartum Intrauterine Contraceptive Device Uptake among Women Delivering in Public Hospitals of South Gondar Zone, Northwest Ethiopia, 2019: An Unmatched Case-Control Study

**DOI:** 10.1155/2021/1757401

**Published:** 2021-02-22

**Authors:** Mandefro Assefaw, Getnet Azanew, Ayenew Engida, Zenebe Tefera, Wondimnew Gashaw

**Affiliations:** ^1^Department of Midwifery, College of Medicine and Health Science, Wollo University, Dessie, Ethiopia; ^2^Department of Midwifery, College of Medicine and Health Science, University of Gondar, Gondar, Ethiopia

## Abstract

**Introduction:**

Integrated use of postpartum intrauterine contraceptive devices with delivery service during the immediate postpartum period is ideal for both women and health-care providers. However, utilization of intrauterine contraceptive devices during the postpartum period was rare and in Ethiopia, with information regarding uptake of postpartum intrauterine contraceptive devices limited.

**Objective:**

Identify determinants of postpartum intrauterine contraceptive devices uptake among women delivering in public hospitals of South Gondar zone, Northwest Ethiopia, 2019.

**Methods:**

An unmatched case-control study was conducted in public hospitals of South Gondar, Ethiopia, from August 1, 2019, to November 10, 2019. A total of 140 cases and 280 controls have actively participated in the study. Five hospitals were selected by simple random sampling. Cases were selected consecutively, whereas two controls for each case were recruited by the lottery method. Pretested questionnaires were used to collect data and it was entered into Epidata version 4.4.2. Logistic regression analysis was used to identify variables associated with the use of outcome and adjusted odds ratio with a 95% confidence interval was used to determine the association between independent and outcome variables.

**Results:**

Completing secondary education (AOR = 4.5, 95%CI 2.3–8.85), having a total number of children of 3–4 (AOR = 3.6, 95%CI 1.25–10.2), having  ≥ 5 (AOR = 4.7, 95%CI 1.5–15.3), attending 3 antenatal care (AOR = 2.8, 95%CI 1.44–5.6), ever hearing about postpartum IUCD (AOR = 6.6, 95%CI 2.7–16.1), and having counseling from health-care provider about a postpartum intrauterine contraceptive device (AOR = 6.2, 95%CI 2.99–12.8) were significantly associated with uptake of the postpartum intrauterine contraceptive. *Conclusion and Recommendation.* Completing secondary education, having 3–4 and ≥5 children, attending three antenatal care, ever hearing about postpartum IUCD, and having counseling from health-care providers about the postpartum intrauterine contraceptive device among women were significantly associated with uptake of an intrauterine contraceptive device after birth. Therefore, it is better to advise women to strictly follow their antenatal care, access to information, and provide counseling.

## 1. Introduction

Postpartum intrauterine contraceptive devices are a provision of IUCD vaginally within 10 minutes after removal of the placenta, called postplacental IUCD, or it can be inserted within 2 days (morning of the first postpartum day before discharge to home) after vaginal delivery, called early postpartum intrauterine contraceptive device. PPIUCD also can be provided during intracesarean after expulsions of the placenta before uterine closure [1].

The immediate postpartum period provides an opportunity for women to initiate effective long-acting contraceptive methods because they are motivated to prevent early subsequent conceptions and have access to health care and this is the ideal time to be certain that the women are not pregnant [1, 2]. The insertion of IUCD during the postpartum period does not affect breast milk, has a lower rate of uterine perforation, does not have other major side effects, and is time-saving and cost-effective, because it can be inserted within few minutes and provided in the same setting with delivery service, and it also reduces crowding of outpatient family planning unit [1, 3]. Therefore, for developing countries, integrated use of PPIUCD with delivery service is the best opportunity to prevent unintended pregnancy. The unmet needs of family planning also can be reduced by providing it immediately after childbirth without a need for repeated visiting of the health-care system and are very convenient for women who will be unable to return for contraception purposes [4]. Despite this advantage, uptake of a postpartum intrauterine contraceptive device during early and immediate postpartum periods is low [5–8]. Reports from the study done at bale zone health facility showed that PPIUCD acceptance was 12.4% [6] and at Sidama zone, PPIUCD utilization was only 21% [7]. The main reason for this low utilization of PPIUCD might be due to the negative contribution of different sociodemographic and obstetric factors.

Even if little researches were conducted in Ethiopia about the postpartum intrauterine contraceptive device, most of them also focused on the proportion of IUCD acceptance and utilization [5–7]. Besides, those researches included factors such as sociodemographic and obstetric related characters; it was not sufficient to show full information about determinants of a postpartum intrauterine contraceptive device, because of sociodemographic characteristic variation throughout different regions of the country.

From most reports of the previous study, the prevalence of postpartum intrauterine contraceptive devices is also rare. Therefore, this study design was appropriate to dig out a full picture of determinants for postpartum intrauterine contraceptive device uptake within a short period. Therefore, the purpose of this study is to fill these gaps by identifying major determinant factors of postpartum intrauterine contraceptive device uptake.

## 2. Methods and Materials

### 2.1. Study Area and Period

An unmatched case-control study was conducted in South Gondar zones public hospitals such as Debretabor general hospital, Mekane-eyesuse primary hospital, Wogeda primary hospital, Addis Zemen primary hospital, and Nifas Mewucha primary hospital from August 1, 2019, to November 10, 2019. South Gondar is one of the zones found in Amhara region and its capital city is Debretabor, which is found 666 km far from Addis Ababa, capital city of Ethiopia. There are eight hospitals found in the zone, which serve 2,609,823 populations. Other maternal and family planning services were given without payment in all public hospitals. Currently, CUT 380 PPIUCD is available in all hospitals.

### 2.2. Study Design

A facility-based unmatched case-control study design was conducted.

### 2.3. Population

#### 2.3.1. Source Population

The source population is all postpartum women who gave birth in public hospitals of South Gondar zone, during the study period.

#### 2.3.2. Study Population

All postpartum women delivering in selected public hospitals during the study period and using a postpartum intrauterine contraceptive device within the first 48 hours following vaginal delivery and intracaesarean after the expulsion of the placenta before uterine closure were considered as cases. However, all postpartum women who gave birth in the same hospitals but did not use postpartum intrauterine contraceptive devices were considered as controls.

### 2.4. Inclusion and Exclusion Criteria

#### 2.4.1. Inclusion Criteria

Postpartum women who gave birth by any mode of delivery in the selected public hospitals were included.

#### 2.4.2. Exclusion Criteria

Women who gave birth by any mode of delivery in selected public hospitals but did not fulfill WHO medical eligibility criteria for PPIUCD during the study period were excluded [9].

### 2.5. Sample Size Determination and Procedure

#### 2.5.1. Sample Size Determination

The sample size for the study was determined with double population proportion formula by using Epi info version 7 statistical software program for an unmatched case-control study. The calculation considered the following assumptions: 95% confidence interval, 80% power, 1 : 2 ratio of cases to controls, with a plan to have another child as the exposure variable, 47.7% of PPIUCD users and 32.8% of nonusers with exposure [7], and 10% nonresponse rate as compensation for both groups. Therefore, 450 postpartum women (150 cases and 300 controls) were included in the study. The sample size for each selected public hospital was proportionally allocated based on previous monthly average PPIUCD utilization.

#### 2.5.2. Sampling Method and Procedures

In south Gondar, there are eight hospitals, and about five (63%) of them were selected by simple random sampling methods. Then the number of PPIUCD utilizations per three months was obtained from each selected public hospital's quarterly report to calculate average monthly cases flow. Cases were identified by asking them whether or not they use IUCD after delivery and by crosschecking their charts and they were selected consecutively, and as soon as cases were identified two controls were selected by simple random methods to increase the power of the study.

### 2.6. Study Variables

#### 2.6.1. Dependent Variables

Uptake of a postpartum intrauterine contraceptive device was a dependent variable.

#### 2.6.2. Independent Variables

(i) Sociodemographic variables are as follows:
    age
    marital status
    religion
    educational status
    residency
    occupation
    education status of the husband
    occupation status of the husband
    family socioeconomic level
(ii) Family planning-related factors are as follows:
    history of family planning use
    type of ever used family planning
    a responsible person decides to use
    having ever heard about postpartum IUCD,
    knowledge about PPIUCD
    attitude towards PPIUCD
(iii) Obstetric, reproductive, and health service-related variables are as follows:
     family size
    parity
    number of live children that women have
    sex composition of live children
    birth interval
    status of current birth
    desire to have children

### 2.7. Operational Definition

 
**Uptake of PPIUCD** is the actual usage of IUCD during the first 48 hours after birth and before discharge to home following any mode of delivery [7].
 
**Counseling** is considered when health-care providers inform women of at least either of the benefits, side effects, duration of pregnancy, and prevention of PPIUCD [7].
 
**Knowledge about PPIUCD** was determined by considering the mean score of correctly answered knowledge assessed questions as a cut point. Postpartum women who answered above or below the mean score of 7 knowledge assessed questions were considered knowledgeable or not knowledgeable, respectively [6].
 
**Attitude**-assessing questions were rated by (disagree, neutral, and agree). Then the response for each item was added by using three Likert scale analyses. Therefore, participants answer above the mean score categorized as having a positive attitude and the reverse categorized as negative attitude [6].

### 2.8. Data Collection Procedures and Instrument

Data were collected using a pretested, semistructured, and interviewer-administered questionnaire. The questionnaire was adapted by reviewing similar researches conducted previously [5–7, 10–12]. The questionnaire involves sociodemographic, obstetric, reproductive, maternal health care, and family planning-related variables. Initially, the questionnaire was prepared in the English version then it was translated into the local language. Five trained BSc midwives and two BSc nurses have participated in data collection and supervisor, respectively. One data collector was assigned to each hospital.

### 2.9. Data Quality Control

To assure the quality of data, the tool was tested by interviewing 5% of postpartum women who gave birth at Gina Mechawocha and Este health center two weeks before the actual data collection and a necessary correction was applied. Half-day training had been given for data collectors and supervisors about the purpose of the study, data collection procedures, and ways of communicating by the principal investigator. Continuous observation of the data collection process and its completeness was assessed every two weeks.

### 2.10. Data Processing and Analysis

At the end of data collection, completeness of data was checked; coding was implemented and entered into Epidata version 4.4.2.1. Statistical software and exported to SPSS version 23 software for further analysis. Descriptive variables were presented using tables and charts.

Bivariate binary logistic regression was used to assess the association between PPIUCD uptake and each factor separately. All variables with *p* value less than or equal to 0.2 in the bivariate logistic regression were entered into the multivariable logistic regression model. Multivariable logistic regression was done by using a backward likelihood ratio method to control potential confounders. Correlation between independent variables was done to check multicollinearity. Finally, the adjusted odds ratio with 95% confidence interval was determined to measure the strength of association, and a *p* value less than 0.05 was used to determine the significant association between factors and outcome variables.

### 2.11. Ethical Consideration

The study obtained ethical clearance from the research review commute of the school of midwifery on behalf of the University of Gondar ethical review board. Written permission was obtained from the responsible body of the South Gondar zone health office and each selected hospital's medical director after approval consent letter was submitted. Verbal informed consent was secured, after the purpose of the study was explained to each study participant.

## 3. Result

### 3.1. Sociodemographic Characters of Respondents

A total of 420 postpartum women (140 cases and 280 controls) were successfully interviewed during the data collection period making a response rate of 93.3%. The median ages of cases and controls were 33 and 28 years, respectively. Regarding the educational status of women, only thirty-six (25.7%) of cases had no formal education. However, nearly half (47.5%) of controls had no formal education. Sixty-five (48.1%) and one hundred forty-five (52%) spouses of cases and controls had no formal education, respectively. Among the participants, twelve (8.6%) cases and forty-five (14.6%) controls were unmarried currently. The majority of cases (97.1%) and controls (98.2%) were Amhara. More than half (55.7%) of cases and nearly half (49.3%) of controls lived in a rural area (Table 1).

### 3.2. Obstetric and Reproductive Characteristics

Both cases and controls have been interviewed regarding their obstetric and reproductive characters. The majority of cases (55%) and controls (57.5%) had a family size of 3–4 followed by ≥ 5 family size for both cases and controls. From respondents, only nine (6.4%) cases and fifty-eight (20.4%) controls did not have a live child at all. Most of the cases (87.9%) and controls (69.3%) were multiparous.

**Figure 1:**
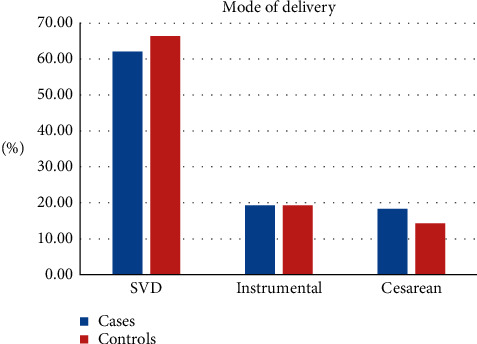
Mode of delivery among respondents delivering in public hospitals of South Gondar zone, Ethiopia, 2019 (*n* = 140 cases and 280 controls).

Concerning the sexual status of live children that women had, eighty-two (62.6%) of the case and hundred twenty-five (56.3%) of controls had both male and female children. Among multiparous women, sixty-four (52%) and one hundred twenty-five (64.4%) cases and controls, respectively, have birth interval greater than 36 months. Among women who planned to have a child, forty-four (35.7%) of cases and seventy-eight (43.6%) of controls desire to have 1–2 children. In addition to this, only two (2.7%) of cases and two (1.1%) of controls want to be pregnant within the first 24 months (Table 2 and Figure 1).

### 3.3. Family Planning-Related Characteristics of Participants

**Figure 2:**
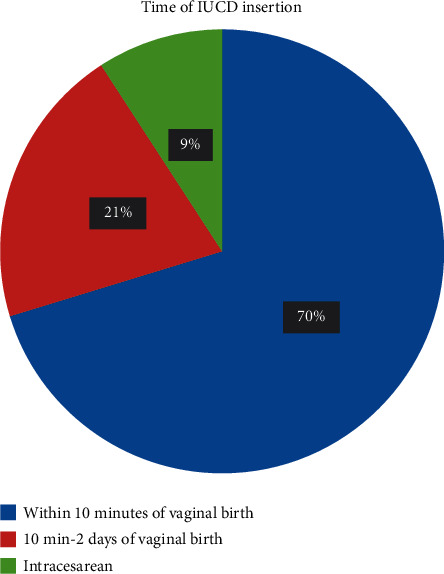
Time of insertion of the postpartum intrauterine contraceptive device among respondents delivering in public hospitals of South Gondar, Ethiopia, 2019 (*n* = 140). Other = includes family, relatives, and friend.

**Figure 3:**
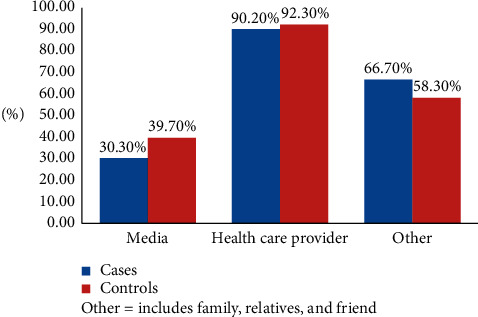
Distribution of information source about family planning among respondents delivering in public hospitals of South Gondar zone, Ethiopia, 2019 (*n* = 132 cases and 156 controls).

The majority of cases (83.6%) and controls (90.4%) used different modern contraceptive methods before the last pregnancy. However, injectable method was the most frequent usable contraceptive method used by both cases, sixty-six (56.4%), and controls, one hundred seventy-three (68.4%), as compared to other methods. One hundred thirty-two (94%) cases and one hundred fifty-six (55.7%) controls heard about the postpartum intrauterine contraceptive device (Table 3, Figures 2 and 3).

### 3.4. Knowledge of Participants about Postpartum IUCD

Eighty-two (58.6%) of cases and one hundred sixteen (41.4%) of controls heard that IUCD can be inserted immediately and 68 (48.6%) of cases and 108 (38.6%) of controls knew that IUCD is a contraceptive method which prevents pregnancy at least 10 years. Sixty (43%) of cases and ninety-three (33%) of controls correctly answered questions about how PPIUCD did not increase the risk of sexually transmitted disease and more than half of cases (54%) and hundred-two (36.4%) of controls knew that IUCD does not interfere with sexual activity. Therefore, the mean of correctly answered knowledge assessed items was 2.92 ± 2SD. Then 28 (20%) of cases and 127 (45.4%) of controls were not knowledgeable and 112 (80%) of cases and about 153 (54.6%) of controls were considered knowledgeable (Table 4).

### 3.5. Level of Attitude towards PPIUCD

Among the total respondents, 55.4% of cases and 68.6% of controls had a negative attitude towards postpartum intrauterine contraceptive devices. However, 44.6% of cases and 31.4% of controls had positive attitudes towards postpartum intrauterine contraceptive device (Table 5).

### 3.6. Determinants of Postpartum Intrauterine Contraceptive Device Uptake

Bivariate binary logistic regression was conducted to detect the association between each independent variable with the outcome variable. Therefore, thirteen variables with *p* value equal to or less than 0.2 were added to the multivariable logistic regression model.

Multivariable logistic regression analysis was done to identify determinants of postpartum intrauterine contraceptive device uptake. Therefore, completing secondary education, having a total number of live children [3, 4] and ≥5, attending three ANC visits during the last pregnancy, having ever heard about the postpartum intrauterine contraceptive device, and having counseling from health-care providers about PPIUCD were significantly associated with uptake of PPIUCD.

In multivariable analysis, women who had completed secondary education were 4.5 times more likely to uptake postpartum intrauterine contraceptive devices than women who had no formal education (AOR = 4.5, 95%CI 2.25–8.9).

Compared to those who had no live child, women who had 3–4 and ≥5 live children were 3.6 times (AOR = 3.6, 95%CI 1.25–10.24) and 4.7 times (AOR = 4.7, 95%CI 1.46–15.3), respectively, more likely to uptake postpartum intrauterine contraceptive device.

The study also identified that women having 3 ANC follow-ups were 2.8 times more likely to uptake postpartum intrauterine contraceptive device as compared to those who did not attend antenatal care during their last pregnancy (AOR = 2.8, 95%CI = 1.1.44–5.6). In addition to this, women who ever heard about PPIUCD were 6.6 times more likely to uptake PPIUCD (AOR 6.6 (95%CI, 2.7–16.1)) as compared to their counterparts.

Finally, the odds of using PPIUCD among women who had got counseling from health-care providers regarding PPIUCD were 6.2 times those among women who did not counsel health-care providers (AOR = 6.2 (95%CI, 2.99–12.85)) (Table 6).

## 4. Discussion

This study identified that factors including completing secondary education, having the number of live children 3–4 and greater or equal to five, attending three ANC visits during last pregnancy, having ever heard about PPIUCD, and having counseling from health-care providers regarding postpartum intrauterine contraceptive device as contraceptive were significantly associated with uptake of the postpartum intrauterine contraceptive device.

This study showed that women who had completed secondary education were more likely to use the postpartum intrauterine contraceptive device as compared to those who had no formal education. This finding contradicts the result reported from the research done in the health facilities of Nigeria [13]. This difference may be due to the difference in the study area and a long time elapse between the two studies. However, the finding was comparable with the result reported from the study done at West Denpasar [14] at Bahirdar [5] and supported by the study done at Bale Zone health facilities [6]. The possible justification for this may be that women who had completed secondary education may have better understanding of the information obtained from health-care providers, media, and other sources. Educated respondents might understand and reject misconceptions, and they may use this method.

The study also observed that women having 3–4 and ≥5 live children were significantly likely to uptake postpartum intrauterine contraceptive devices than those who did not have children previously. The finding was supported by similar results from a study done at the private health facilities of Nigeria [13]. The possible explanation for this finding is that women who have a large number of children may be motivated to prevent further pregnancy to limit her family size.

This study also identified that women who had 3 antenatal care follow-ups throughout their last pregnancy were more likely to use the postpartum intrauterine contraceptive device as compared to those who did not attend ANC. The result is consistent with the study done at bale zone health facilities, Ethiopia [6]. This may be due to the fact that focused antenatal care recommends counseling about contraceptive methods during antenatal care follow-up and that should start from their third visit and it is the ideal time to counsel women about family planning to use during the postpartum period [15]. Therefore, the increased frequency of ANC follow-ups to three times may allow pregnant women to access an opportunity for discussion about the postpartum intrauterine contraceptive device as other methods.

The study observed that women who ever heard about the postpartum intrauterine contraceptive device were more likely to use them than their counterparts. The result is consistent with the study done at Sidama public health facility [7], since, from EDHS 2016 reports, still, the overall utilization of intrauterine contraceptive devices is rare, less than 2% [9], which may be attributed to lack of information about this method as compared to other contraceptive methods.

Finally, the study also identified that women who had got counseling from health-care providers about postpartum intrauterine contraceptive devices significantly contribute to the uptake of the postpartum intrauterine contraceptive device. This finding is supported by the study done at four countries [16], Bhaktapur, Nepal [17], and in line with the study done at tertiary hospital Odisha, India [12], and the public health facility of Bale zone Ethiopia [6] and health center at Bahirdar, Ethiopia [5]. The possible justification could be that counseling by health-care providers may allow women to get accurate information about PPIUCD that can change their attitudes and behaviors by avoiding rumors and misconceptions, which may hinder the acceptance of postpartum intrauterine contraceptive devices to use. Therefore, avoiding this behavior through good and free counseling may motivate women to use the intrauterine contraceptive device during their postpartum period immediately after delivery.

## 5. Limitation of Study

This research was conducted only in public hospitals, the finding could not be used to generalize women who gave birth at home, health centers, and private health institutions, and there is no information regarding qualitative data. Another limitation of the study was that women might be discharged from the hospital before being interviewed so it was needed to actively follow and collect data from them before discharge.

## 6. Conclusion

The present study identified that completing secondary education, having a number of 3–4 and greater or equal to five children, attending three ANC visits during the last pregnancy, having ever heard about the postpartum intrauterine contraceptive device, and having counseling from health-care providers about postpartum IUCD as part of the contraceptive method were significantly associated with uptake of a postpartum intrauterine contraceptive device. The study recommends emphasizing the dissemination of information about the postpartum intrauterine contraceptive devices to women, encouraging women to attend at least secondary education, counseling women on postpartum IUCD during any contact, and motivating women to attend their ANC follow-ups.

## Figures and Tables

**Table 1:** Sociodemographic characters of respondents who gave birth in South Gondar public hospitals, Northwest Ethiopia, 2019.

Characteristics	Uptake of PPIUCD	
	Cases *n* = 140 (%)	Controls *n* = 280 (%)
Age of women in a year		
15−24	24 (17.1%)	68 (24.3%)
25−34	59 (42.1%)	161 (57.5%)
35−44	57 (40.7%)	51 (18.2%)

Educational status of women		
No formal education	36 (25.7%)	133 (47.5%)
Primary	26 (18.6%)	59 (21.1%)
Secondary	58 (41.4%)	47 (16.8%)
Higher education	20 (14.3%)	41 (14.6%)

Occupational status of women		
Housewife	79 (56.4%)	188 (67.1%)
Daily laborer	18 (12.9%)	21 (7.5%)
Merchant	7 (7.7%)	16 (5.7%)
Government employee	15 (10.7%)	32 (11.4%)
Private employee	9 (6.4%)	7 (2.5%)
Student	12 (8.6%)	16 (5.7%)

Husband occupational status		
Farmer	65 (47.8%)	125 (46.3%)
Daily laborer	14 (11%)	25 (9.3%)
Merchant	15 (10%)	48 (17.8%)
Government employee	22 (16.2%)	51 (18.9%)
Private employee	9 (6.6%)	12 (4.5%)
Student	10 (7.4%)	9 (3.3%)

Family wealth index		
Lowest	50 (35.7%)	121 (43.2%)
Middle	55 (39.3%)	34 (12.1%
Highest	35 (25%)	125 (44.6%)

*a* = Oromo, Tigre, *b* = Muslim, Protestant, and Catholic, *c* = single, separated, divorced, and widowed.

**Table 2:** Obstetric, reproductive, and maternal health-care characteristics of respondents delivering in public hospitals of South Gondar zone, Ethiopia, 2019 (*n* = 140 cases and 280 controls).

Characteristics	Uptake of PPIUCD	
	Cases *n* = 140 (%)	Controls *n* = 280 (%)
Total family size		
≤2	12 (8.6%)	45 (16.1%)
3−4	77 (55%)	161 (57.5%)
≥5	51 (36.4%)	74 (26.4%)

No. of total live children women have		
No children at all	9 (6.4%)	58 (20.4%)
1−2	48 (34.3%)	137 (48.9%)
3−4	51 (36.4%)	47 (16.8%)
≥5	32 (22.9%)	38 (13.6%)

Sex status of live children (**n1** = **131**, **n2** = **222**)		
Only female	16 (12.2%)	35 (15.8%)
Only male	33 (25.2%)	62 (27.9%)
Both male and female	82 (62.6%)	125 (56.3%)

Parity of respondents		
Primipara	17 (12.1%)	86 (30.7%)
Multipara	123 (87.9%)	194 (69.3%)

Birth interval by months (**n1** = **123**, **n2** = **194**)		
Less than 24	46 (37.4%)	47 (24.2%)
26−36	13 (10.6%)	22 (11.3%)
Greater than 36	64 (52%)	125 (64.4%)

Did you have the plan to have a child?		
Yes	73 (52.1%)	179 (63.9%)
No	59 (42.1%)	69 (24.6%)
Undecided	8 (5.7%)	32 (11.4%)

No. of children you plan to have (**n1** = **73**, **n2** = **179**)		
1−2	44 (35.7%)	78 (43.6%)
3−4	25 (30.5%)	78 (43.6%)
≥5	4 (5.4%)	23 (12.8%)

Sex status of children women desire (**n1** = **73**, **n2** = **179**)		
Only male	9 (12.3%)	19 (10.6%)
Only female	5 (6.8%)	6 (3.4%)
Both	59 80.8%)	154 (86%)

Decision maker for planned children (**n1** = **73**, **n2** = **179**)		
Myself	20 (27.4%)	54 (30.1%)
Husband	31 (41.3%)	58 (32.4%)
Joint decision	22 (30.1%)	67 (37.4%)

Time to have children by month (**n1** = **73**, **n2** = **179**)		
Less than 24	2 (2.7%)	2 (1.1%)
24−36	22 (30.1%)	100 (55.9%)
Greater than 36	49 (67.1%)	77 (43%)

Status of current birth		
Planned	123 (87.9%)	233 (83.2%)
Unplanned	17 (12.1%)	47 (16.8%)

Number of ANC visits		
Did not attend	28 (20%)	96 (34.3%)
Attended one times	8 (5.7%)	30 (10.7%)
Attended two times	9 (6.4%)	47 (16.8%)
Attended three times	68 (18.6%)	64 (22.9%)
Attended ≥4 times	27 (19.3%)	43 (15.4%)

Counseling		
Yes	126 (90%)	121 (43.2%)
No	14 (10%)	159 (56.8%)
Partner involvement for discussion (**n1** = **126**, **n2** = **121**)		
Yes	65 (51.6%)	50 (41.3%)
No	61 (48.4%)	71 (58.7%)

Time of counseling (**n1** = **126**, **n2** = **121**)		
During ANC	34 (27%)	13 (11.2%)
During LFSOL	23 (18.3%)	15 (12.9%)
During PNC	28 (22.2%)	34 (20.7%)
Multiple counseling a	41 (32.5%)	59 (55.2%)

**a** = woman who had counseled more than one time, **n1** = cases and **n2** = controls , **ANC** = antenatal care, **LFSOL** = latent first stage of labor, **PNC** = postnatal care.

**Table 3:** Family planning-related characteristics of respondents delivering in selected public health hospitals of South Gondar zone, Ethiopia, 2019 (*n* = 140 cases and 280 controls).

Characteristics	Uptake of PPIUCD	
	Cases *n* = 140 (%)	Controls *n* = 280 (%)
Ever used family planning before the last PX		
Yes	117 (83.6%)	253 (90.4%)
No	23 (16.4%)	27 (9.6%)

Type of modern family planning ever used^a^		
Pill	37 (31.6%)	69 (27.3%)
Injectable	66 (56.4%)	173 (68.4%)
Implants	43 (36.8%)	67 (26.5%)
IUCD	15 (12.8%)	7 (27.7%)

Who decides to ever use family planning?		
Myself	33 (28.2%)	82 (32.4%)
Husband	10 (8.5%)	14 (5.5%)
Other (health-care provider, family)	74 (63.2%)	157 (60.5%)

Ever heard about PPIUCD		
Yes	132 (94%)	156 (55.7%)
No	8 (5.7%)	124 (44.3%)

**a** = more than one answer is possible, **px** = pregnancy, **IUCD** = intrauterine contraceptive device. **PPIUCD** = postpartum intrauterine contraceptive device.

**Table 4:** Knowledge about PPIUCD among respondents delivering in public hospitals of South Gondar zone, Northwest Ethiopia, December 2019 (*n* = 140 cases and 280 controls).

Variable	Uptake of PPIUCD			
	Cases *n* = 140 (%)		Controls *n* = 280 (%)	Total
IUCD can be inserted immediately after delivery	Yes	82 (58.6%)	116 (41.4%)	198
	No	58 (41.4%)	164 (58.6%)	222

IUCD prevents pregnancy for at least 10 years	Yes	68 (48.6%)	108 (38.6%)	176
	No	72 (51.4%)	172 (61.4%)	244

IUCD is a contraceptive method that can be put in the uterus.	Yes	75 (53.6%)	99 (35.4%)	174
	No	65 (46.4%)	181 (64.6%)	246

IUCD does not increase the risk of STI	Yes	60 (42.9%)	93 (33.2%)	153
	No	80 (57.1%)	187 (66.8%)	267

IUCD does not interfere with sexual activity	Yes	76 (54.3%)	102 (36.4%)	178
	No	64 (45.7%)	178 (63.6%)	242

Pregnancy is immediately reversible after removal of IUCD	Yes	75 (53.6%)	102 (36.4%)	177
	No	65 (46.4%)	178 (63.6%)	243

IUCD does not cause cancer of genital tract	Yes	69 (49.6%)	91 (32.5%)	160
	No	71 (51,4%)	189 (67.5%)	260

**Table 5:** Participants attitudes towards PPIUCD among women who gave birth at public hospitals of South Gondar, Ethiopia, 2019 (*n* = 140 cases and 280 controls).

Variable	Category	Uptake of PPIUCD	
		Cases *n* = 140 (%)	Controls *n* = 280 (%)
Insertion and removal PPIUCD did not have severe pain	Disagree	71 (50.7%)	112 (40%)
	Neutral	62 (44.3%)	95 (33.9%)
	Agree	7 (5%)	73 (26.1%)

PPIUCD should not cause irregular vaginal bleeding	Disagree	62 (44.3%)	106 (37.9%)
	Neutral	69 (49.3%)	99 (35.4%)
	Agree	9 (6.4%)	75 (26.8%)

PPIUCD should not cause loss of privacy	Disagree	57 (40.7%)	89 (31.8%)
	Neutral	76 (54.3%)	106 (37.9%)
	Agree	7 (5%)	85 (30.4%)

PPIUCD should not restrict normal activity	Disagree	55 (39.3%)	81 (28.9%)
	Neutral	78 (55.7%)	120 (42.9%)
	Agree	7 (5%)	79 (28.2%)

PPIUCD should impair future fertility	Disagree	48 (34.3%)	86 (30.7%)
	Neutral	78 (55.7%)	112 (40%)
	Agree	14 (10%)	82 (29.3%)

PPIUCD should not rust inside the womb	Disagree	52 (37.1%)	88 (31.4%)
	Neutral	72 (51.4%)	101 (36.1%)
	Agree	16 (11.4%)	91 (32.5%)

PPIUCD should not be stuck on the fetal head if px occur during IUCD in-situ	Disagree	62 (44.3%)	92 (32.9%)
	Neutral	52 (37.1%)	92 (32.9%)
	Agree	26 (18.6%)	96 (34.3%)

PPIUCD is not for use by only older women but also for all.	Disagree	55 (39.3%)	104 (37.1%)
	Neutral	64 (45.7%)	89 (31.8%)
	Agree	21 (15%)	87 (31.1%)

PPIUCD should not move around the body	Disagree	68 (48.6%)	109 (38.9%)
	Neutral	49 (35%)	85 (30.4%)
	Agree	23 (16.4%)	86 (30.7%)

PPIUCD should not damage womb	Disagree	61 (43.6%)	107 (38.2%)
	Neutral	55 (39.3%)	93 (33.2%)
	Agree	24 (17.1%)	80 (28.6%)

**Table 6:** Bivariate and multivariable logistic regression analysis of determinant factors for the uptake of PPIUCD among women delivering in public hospitals of South Gondar zone, Ethiopia, 2019 (*n* = 140 cases and 280 controls).

Variables	Uptake of PPIUCD		COR (95%CI	AOR (95%CI
	Cases (*n* = 140)	Controls (280)		
Age of women by years				
15−24	24	68	1	1.00
25−34	59	161	1.04 (0.6–1.81)^*∗*^	0.8 (0.37–1.60)
35−44	57	51	3.2 (1.7–5.77)	2.1 (0.84–5.43)

Marital status				
Unmarried	12	41	1.00	1.00
Married	128	239	1.8 (0.93–3.6)^*∗*^	1.93 (0.74–5.0)

Women educational status				
No formal education	36	133	1.00	1.00
Primary	26	59	1.63 (0.9–2.94)^*∗*^	1.2 (0.6–2.57)
Secondary	58	47	4.56 (2.7–7.77)^*∗*^	**4.5** (**2.3**−**8.85**)^*∗∗*^
Higher	20	41	1.8 (0.94–3.45)^*∗*^	1.7 (0.76–3.98)

Family size				
≤2	12	45	1	1.00
3–4	77	161	1.8 (0.9–3.58)^*∗*^	1.4 (0.53–3.4)
≥5	51	74	2.6 (1.3–5.36)^*∗*^	1.14 (0.42–3.1)

Total no. of live children				
No child at all	9	57	1	1.00
1−2	48	137	2.2 (1.0–4.82)^*∗*^	1.8 (0.69–4.7)
3−4	51	47	6.9 (3.1–15.4)^*∗*^	**3.6** (**1.25**−**10.2**)^*∗∗*^
≥5	32	38	5.3 (2.3–12.4)^*∗*^	**4.7** (**1.5**−**15.3**)^*∗∗*^

Parity				
Primipara	17	86	1.00	1.00
Multipara	123	194	3.2 (1.8–5.66)^*∗*^	0.84 (0.3–2.5)

Plan to have children				
No	59	69	1	
Undecided	8	32	0.3 (0.13–0.68)^*∗*^	0.45 (0.14–1.42)
Yes	73	179	0.5 (0.3–0.74)^*∗*^	0.5 (0.27–1.03)

Frequency of ANC follow-up				
Did not attend	28	96	1.00	1.00
Attended one	8	30	0.9 (0.4–2.22)	0.5 (0.16–1.54)
Attended two	9	47	0.66 (0.29–1.5)	0.3 (0.11–0.84)
Attended three	68	64	3.64 (2.1–6.3)^∗^	**2.8 (1.44**−**5.6)**^*∗∗*^
Attended ≥4	27	43	2.2 (1.14–4.1)^*∗*^	2.05 (0.9–4.8)

Ever use of family planning				
No	23	27	1	1.00
Yes	117	253	0.5 (0.3–0.99)^*∗*^	0.5 (0.22–1.2)

Ever heard about PPIUCD				
No	8	124	1	1.00
Yes	132	156	13.1 (6.2–27.8)^*∗*^	**6.6 (2.7**−**16.1)**^*∗∗*^

Knowledge about PPIUCD				
Not knowledgeable	28	127	1.00	1.00
Knowledgeable	112	153	3.3 (2.1–5.35)^*∗*^	1.64 (0.8–3.3)

Attitude towards PPIUCD				
Negative attitude	96	155	1.00	1.00
Positive attitude	44	125	0.6 (0.4–0.87)^*∗*^	1.2 (0.6–2.2)

Counseled by a health-care provider				
No	14	159	1	1.00
Yes	126	121	11.8 (6.5–21.6)^*∗*^	**6.2 (2.9**−**12.8)**^*∗∗*^

^*∗*^(*p* < 0.2) in bivariate, 1 = reference group ^*∗∗*^ = statically significant at *p* < 0.05 in multivariate, Hosmer Lemeshow model fit = 0.95.

## Data Availability

The data sets generated during this study are available from the corresponding author upon reasonable request.
